# Effect of Height of Fall on Mortality in Patients with Fall Accidents: A Retrospective Cross-Sectional Study

**DOI:** 10.3390/ijerph17114163

**Published:** 2020-06-11

**Authors:** Ting-Min Hsieh, Ching-Hua Tsai, Hang-Tsung Liu, Chun-Ying Huang, Sheng-En Chou, Wei-Ti Su, Shiun-Yuan Hsu, Ching-Hua Hsieh

**Affiliations:** 1 Department of Trauma Surgery, Kaohsiung Chang Gung Memorial Hospital and Chang Gung University College of Medicine, Kaohsiung 88301, Taiwan; hs168hs168@gmail.com (T.-M.H.); tsai1737@cloud.cgmh.org.tw (C.-H.T.); htl1688@yahoo.com.tw (H.-T.L.); junyinhaung@yahoo.com.tw (C.-Y.H.); athenechou@gmail.com (S.-E.C.); s101132@adm.cgmh.org.tw (W.-T.S.); ah.lucy@hotmail.com (S.-Y.H.); 2 Department of Plastic Surgery, Kaohsiung Chang Gung Memorial Hospital and Chang Gung University College of Medicine, Kaohsiung 88301, Taiwan

**Keywords:** height of fall, fall accident, injury severity score (ISS), mortality, ground-level falls (GLF)

## Abstract

Background: Accidental falls are a common cause of injury and deaths. Both ground-level falls (GLF) and non-GLF may lead to significant morbidity or mortality. This study aimed to explore the relationship between height of falls and mortality. Method: This is a retrospective study based on the data from a registered trauma database and included 8699 adult patients who were hospitalized between 1 January 2009 and 31 December 2017 for the treatment of fall-related injuries. Study subjects were divided into three groups of two categories based on the height of fall: GLF (group I: < 1 m) and non-GLF (group II: 1–6 m and group III: > 6 m). The primary outcome was in-hospital mortality. The adjusted odds ratio (AOR) of mortality adjusted for age, sex, and comorbidities with or without an injury severity score (ISS) was calculated using multiple logistic regression. Results: Among the 7001 patients in group I, 1588 in group II, and 110 in group III, patients in the GLF group were older, predominantly female, had less intentional injuries, and had more pre-existing comorbidities than those in the non-GLF group. The patients in the non-GLF group had a significantly lower Glasgow Coma Scale (GCS), a higher injury severity score (ISS), worse physiological responses, and required more procedures performed in the emergency department. The mortality rate for the patients in group I, II, and III were 2.5%, 3.5%, and 5.5%, respectively. After adjustment by age, sex, and comorbidities, group II and group III patients had significantly higher adjusted odds of mortality than group I patients (AOR 2.2, 95% CI 1.64–2.89, *p* < 0.001 and AOR 2.5, 95% CI 1.84–3.38, *p* < 0.001, respectively). With additional adjustment by ISS, group II did not have significantly higher adjusted odds of mortality than group I patients (AOR 1.4, 95% CI 0.95–2.22, *p* = 0.082), but group III patients still had significantly higher adjusted odds of mortality than group I patients (AOR 10.0, 95% CI 2.22–33.33, *p* = 0.002). Conclusion: This study suggested that patients who sustained GLF and non-GLF were distinct groups of patients, and the height of fall did have an impact on mortality in patients of fall accidents. A significantly higher adjusted odds of mortality was found in the GLF group than in the non-GLF group after adjusting for age, sex, and comorbidities.

## 1. Introduction

Falls are a common reason for trauma care emergency department (ED) visits in all age groups [1]. According to the World Health Organization, falls are the second leading cause of accident or unintentional injury deaths in the world [2]. In the United Kingdom, a multicenter study of 31,419 traumatic ED admissions demonstrated that fall accidents accounted for 55.3% of ED visits and 3.5% of deaths [3]. In Japan, falls accounted for 41.4% of ED admissions and led to 4.4% of 28-day mortality, according to a study on 80,813 trauma patients via Trauma Data Bank [4]. Our prior study on 16,548 hospitalized patients from the Trauma Registry System of a trauma center in Taiwan revealed that falls accounted for 30.3% of ED admissions and caused 4.5% of in-hospital mortality [5]. This shows that fall accidents cause a considerable portion of traumatic injuries and non-negligible fatality.

As height affects the velocity of a fall, theoretically, a fall from a higher height causes more severe injuries [6]. Especially, a fall >6 m (m) is counted as high energy trauma and a transfer to a trauma center is recommended [7]. However, the factors affecting mortality in falls are complex and data on the height of fall affecting the mortality of fall accidents remain inconsistent [8,9,10,11,12,13]. A prehospital retrospective study including injured or deceased adult patients by Dickinson et al. [10] demonstrated that the height of fall is a significant predictor of mortality with the possibility of dying increased by 23% for every meter fallen. A study on patients who fell from a height >3 m by Lapostolle et al. [9] reported that the height of fall was significantly correlated with a higher odds of mortality. However, there are also studies amongst the literature that suggest that the height of fall is not a reliable predictor of injury, severity or mortality. Katz et al. [12] revealed that mortality was associated with the intention rather than the height of fall. Goodacre et al. [14] demonstrated that in fall accidents, the severity of injury increases with increasing age and head trauma, whereas the height of fall cannot predict the mortality or major injury with an injury severity score (ISS) >15. A retrospective study on 66 patients of falls from heights >6 m by Liu et al. [8] revealed that the only independent prognostic factor is severe head injury, which is expressed with the abbreviated injury scale (AIS) score as ≥4 in the head region. In addition, the injury severity estimated by ISS cannot predict the height of falls accurately [15].

Furthermore, although ground-level falls (GLF) are often deemed an innocuous low energy mechanism of injury, a mortality of 3.2% was found in a study of 57,302 patients with GLF [16]. Some studies showed that such low impact falls are often underestimated and may cause significant injuries with considerable demands on the system of trauma care [3,17]. These patient populations were recommended to be transported to trauma centers, because they continued to have a 2.8–8% mortality rate. Moreover, the study on 8111 adults by Wang et al. indicated that compared to high levels of falls, such GLF can predict the long-term mortality independently of age, sex, comorbidity and injury severity of the patients [18]. Therefore, despite previous studies [8,9,10,11,12,13] which addressed the relationship between the height of fall and mortality, controversy in the conclusion prompted us to conduct a study to evaluate the effect of the height of fall on mortality based on registered trauma data from a level I trauma center.

## 2. Methods

### 2.1. Ethical Statement

This study was conducted at the Kaohsiung Chang Gung Memorial Hospital, a 2686-bed facility and a level I regional trauma center that provides care to trauma patients primarily from South Taiwan. Approval for this study was obtained before its initiation from the hospital’s institutional review board with approval number 201801328B0.

### 2.2. Study Population

Adult patients aged ≥20 years and hospitalized between 1 January 2009 and 31 December 2017 for the treatment of fall-related injuries were included in this study. The cut-off age of 20 was arbitrarily selected to be an adult because in Taiwan, there is a legal requirement of supervisors to proceed healthcare-related tasks such as admission, invasive procedures and operation until the patient turns 20 years old. All hospitalized patients by all trauma causes should be registered into the trauma registry system. The trauma registry system classifies fall heights in three categories, which facilitates statistical interpretation. This classification of fall heights was made following a review of several papers that defined GLF as a fall from a height of less than 1 m [19]; a low-level fall as a fall from a height between 1m and 6 m; a higher level fall as a fall from higher than 6 m [7]. GLF is often deemed a low-energy mechanism of injury and not a recommended triage criterion for trauma team activation. Contrastingly, in cases of falls from higher than 6 m (which is used as the threshold energy of the cut-off point for trauma triage criteria), a transfer to a trauma center is recommended by the American College of Surgeons [7]. Patients with incomplete or missing data (*n* = 0) or those who were dead at scene were excluded from the study.

### 2.3. Data Collection and Outcome Measures

There is a retrospective study of patients’ medical data retrieved from the Trauma Registry System of our hospital [20,21,22], which serves over three million people in the southern area of Taiwan and has another two level I trauma centers. There were around more than 17,000 ED visits and 3600 hospitalizations per year. We collected the relevant information including intent (suicidal jumps) or lack thereof to fall (accident falls or escaped attempts); the height of fall in meters (m); physiological signs on arrival to ED; systolic blood pressure (SBP); respiratory rate (RR); Glasgow Coma Scale (GCS) on which patients with a GCS of 3–8 were deemed to have several head injuries, those with a GCS of 9–12 a moderate head injury, and those with a GCS of 13–15 as mild head injury; AIS in six body regions; ISS (patients with an ISS of 1–15 deemed as minor to moderate trauma, an ISS of 16–24 as a severe injury, and an ISS ≥25 as a critical injury); procedures performed at ED, including endotracheal intubation, thoracotomy, and blood transfusion; associated injuries in body regions; length of stay (LOS) in hospital; requirement for intensive care unit (ICU) admission; and in-hospital mortality. Moreover, data were collected on pre-existing comorbidities and chronic diseases including diabetes mellitus (DM), hypertension (HTN), coronary artery disease (CAD), congestive heart failure (CHF), cerebral vascular accident (CVA), and end stage renal disease (ESRD).

### 2.4. Statistical Analysis

In this study, patients were divided into two groups: GLF and non-GLF. The subjects with GLF was indicated as group I, meaning that the patients had a fall from a height less than 1 m. The subjects with non-GLF were grouped in two categories—group II, which included falls from heights between 1 m and 6 m, and group III, which included falls occurring from heights higher than 6 m. The differences between non-GLF (groups II and III) and GLF (group I) were compared based on the primary outcome of in-hospital mortality and the secondary outcomes, which were hospital LOS and rates of ICU admissions. The patient characteristics are summarized as mean ± standard deviation, median with interquartile range (GCS and ISS), or frequency (%) as appropriate. Descriptive statistics were obtained by calculating the mean and standard deviation for continuous variables and relative frequencies for categorical variables. These groups were compared using the chi-square test for categorical variables and the analysis of variance (ANOVA) with post hoc tests for continuous variables. The adjusted odds ratio (AOR) of mortality adjusted for age, sex, and comorbidities calculated using multiple logistic regression are presented as 95% confidence intervals (CIs). A *p* value <0.05 was considered statistically significant for all comparisons.

## 3. Results

### 3.1. Study Populations

**Figure 1:**
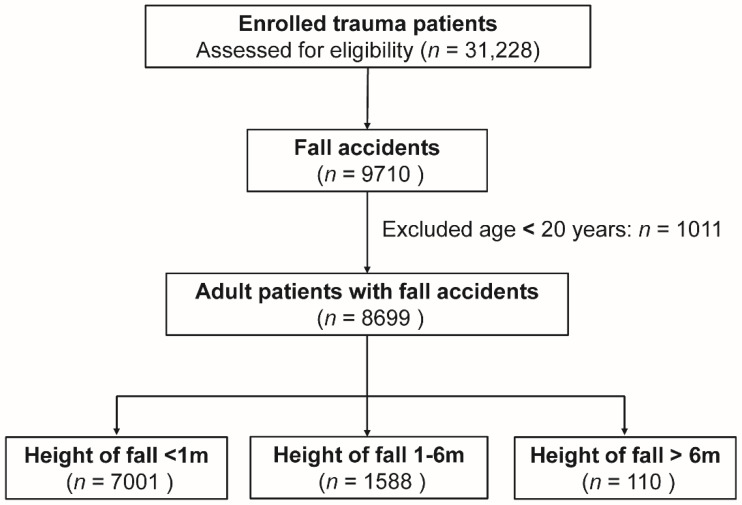
Flow chart illustrating the inclusion of patients with a fall from the height of <1 m (m), 1–6 m, and >6 m.

Of the 31,228 enrolled trauma patients, 9710 patients sustained a fall accident. Those aged <20 years were excluded (*n* = 1011), leaving a total of 8699 patients being the subjects of this study. These patients were divided into three groups according to the height of fall (<1 m, 1–6 m, and >6 m), with 7001 patients in group I, 1588 patients in group II, and 110 patients in group III (Figure 1).

### 3.2. Patient and Injury Characteristics

As shown in Table 1, the patients who had sustained GLF (group I) were older, predominantly female, had less intention of injury, and had more pre-existing comorbidities than those who had sustained non-GLF (group II and III). There were significantly higher rates of intentional attempts in group II and group III than in group I. In addition, there were significantly lower rates of almost all recorded comorbidities in group II and group III when compared to group I. As shown in Table 2, the patients in group II and group III had a significantly lower GCS and higher ISS than those in group I, with more patients in group II and group III presenting severe conscious loss, a GCS between 3–8, as well as severe (ISS between 16–24) and critical injuries than those in group I. Compared to group I patients, the rates of injuries to the body regions with AIS ≥ 2 were significantly higher in the head/neck, face, thorax and abdomen regions in patients of group II and group III. The patients in group II (average 10.6 days) and group III (21.2 days) had a significantly longer hospital stay than those in group I (8.2 days). The patients in group II (23.2%) and group III (58.2%) had a significantly higher rate of ICU admission than those in group I (14.7%). There were significantly higher odds of mortality in group II patients than those in group I (OR 1.4, 95% CI 1.03–1.90, *p* = 0.033), but such significant difference was not found between the patients in group III and group I (OR 2.2, 95% CI 0.96–5.10, *p* = 0.056). After adjustment by age, sex, and comorbidities, group II and group III patients had significantly higher adjusted odds of mortality than group I patients (AOR 2.2, 95% CI 1.64–2.89, *p* < 0.001 and AOR 2.5, 95% CI 1.84–3.38, *p* < 0.001, respectively) (Table 1). Under the adjustment by age, sex, comorbidities, and additional ISS, group II did not have significantly higher adjusted odds of mortality than group I patients (AOR 1.4, 95% CI 0.95–2.22, *p* = 0.082); however, with additional adjustment by ISS, group III patients still had significantly higher adjusted odds of mortality than group I patients (AOR 10.0, 95% CI 2.22–33.33, *p* = 0.002).

### 3.3. Physiological Responses and Procedures Performed

Regarding the physiological responses and procedures performed at the ED (Table 3), there were significantly higher incidences of GCS < 13, SBP < 90 mmHg, and RR < 10 or >29 beats/min in group II and group III, than in group I (all *p* < 0.001). Furthermore, there were significantly higher rates of patients receiving intubation, thoracotomy, and blood transfusion in group II and III than in group I (all *p* < 0.001). In the ED, the conditions of the patients in group II and III were rather critical compared to those of the patients in group I.

### 3.4. Associated Injuries on Body Regions

The rate of associated injuries on body regions are presented in Table 3. As observed, with the exception of extremity injury, there were significantly higher rates of injuries sustained in almost all body regions including head, maxillofacial, thoracic, and abdominal areas in group II and III patients than group I patients (Table 4). Notably, there was a significantly lower rate of femoral fracture in group II and group III when compared to group I (10.9% vs. 39.7%, *p* < 0.001 and 16.4% vs. 39.7%, *p* < 0.001, respectively).

## 4. Discussion

This study suggested that those patients who sustained GLF (group I) and non-GLF (group II and III) were distinct groups of patients, with patients who sustained GLF being older, predominantly female, with less intentional injuries and more pre-existing comorbidities. This characteristic reasonably reflected that there were significantly more incidences of femoral fracture in GLF than in non-GLF. On the other hand, our results showed the middle-aged men seemed predominant in the non-GLF groups. This may be due to the fact that falls from great heights usually occur accidently and are often work-related. Son et al. [23] conducted a study with 2147 victims attending the ED due to occupational injuries, and they reported a mean age of 46 years old for patients of fall-related occupation injuries, of which, 32% were construction site-related injuries and 70% injuries occurred during regular working hours from 09:00 to 18:00. Likewise, Jagnoor et al. [24] reported that most worked-related falls were in the working age (22% in the age group of 15–34 years and 17% in the age of 35–59 years) and amongst males in regard to falls at a construction sites. Similarly, our result implied that the majority of the elderly female patients sustained GLF upon walking or with movement, and that the majority of the male adults sustained non-GLF due to more rigorous activity. The results also demonstrated that those patients with non-GLF were more severely injured, had a significantly worse physiological response, and required more resuscitation procedures than those with GLF. Although significantly higher odds of mortality were only found in group II and not in group III, when compared with those in group I, it could even be suggested that falls from higher heights may cause more severe injuries. However, the difference in mortality rate between group III and group I may be reduced because the patient populations with GLF and non-GLF are different. Moreover, after adjusting for age, sex, and comorbidities, a significantly higher adjusted odds of mortality was seen in group III when compared to that of group I.

Based on our study, the height from fall was found to be an independent predictor of mortality, which conformed to those of previous studies by Lapostolle et al. [9] in 2005 and Dickinson et al. [10] in 2012, but in contrast to the studies of Liu et al. [8] and Katz et al. [12]. Additionally, a 4-year study with an evaluation of 2252 trauma patients of falls suggested that the height of fall had statistically significant effects on mortality in univariate analysis, but it failed to maintain this significance after multivariate analysis [11]. According to the above-mentioned studies, the conclusions are inconsistent. Apart from the complex factors of fall related injuries, the most important reasons for the contradictory results may be due to the differences in the studied populations (such as different inclusion criteria of height of falls or type, severity, site of injury, out-of-hospital deaths, etc.).

Age, sex, and comorbidities are different variables but important determinants of outcomes after the injury among those who had GLF and non-GLF. After high falls, age is another independent prognostic factor for mortality [9,13]. Older people or patients with comorbidities had a worse result than their younger counterparts [9,13]. Elderly women are more likely to attend the ED with a fall injury [4,25]. One study [25] of 15,662 adult patients and another study [4] of 15,207 adult patients with GLF demonstrated that old female populations had significantly higher frequencies of lower limbs injury (AIS ≥ 3). This could be explained by the fact that older women frequently suffered from postmenopausal osteoporosis [26]. Additionally, high-heeled shoes may cause a negative effect that transports up the low limb, leading to a higher risk of fall, fracture, and ankle sprain [27]. This may be another risk factor contributing to GLF in women. Furthermore, both studies [4,25] also demonstrated that the older female population had significantly lower incidences and risk of mortality. A study on 80,813 trauma patients concluded that males experiencing GLF had a significantly greater 28-day mortality (AOR 1.34, 95% CI 1.19–1.52, *p* < 0.0001) after adjusting for severity [4]. In addition, those who were males and had pre-existing conditions experienced poorer outcomes in low falls [28]. In contrast, it was reported by Kennedy et al. [3] that female sex was associated with worse prognosis after low falls. Nonetheless, in this study we tried to attenuate the confounding effects of the baseline patient characteristics by adjusting for age, sex, and comorbidities, and we found that there was a significantly higher adjusted odds of mortality in patients with non-GLF than in those with GLF.

Another contention that has been addressed is the relationship between height of fall and injury severity. Goodacre et al. [14] deemed that height is a poor indicator of injury severity in high falls. Furthermore, some studies found the possibility for low falls to cause severe outcomes [16,17,29]. However, most studies on free falls have reported that the height of fall significantly correlates with injury severity [9,10,13,30], and our results also support this correlation. Atanasijevic et al. [30] performed a retrospective analysis of 660 cases of fatal falls from a height and inferred that the frequency and extent of injuries of various body regions and organs are in correlation with the height of fall. Similarly, Petaros et al. [31] concluded that the height of free fall was the major factor affecting fracture patterns, and that it significantly correlates with the number of injury regions. Lau et al. [32] indicated that it is feasible to construct mathematical models associated with the height of fall to the severity and extent of injuries sustained. Dickinson et al. [10] suggested that chest or head injuries significantly increased the possibility of mortality after a fall from height. In their study, the odds of mortality showed a 2.47-fold increase for head injury and a 2.29-fold increase for chest injury for every meter fallen. Similarly, Içer et al. [11] reported that subarachnoid hemorrhage and hemothorax are the most independent risk factors affecting mortality in fall. Considering that a severe trauma of the head or chest may necessitate intubation or thoracotomy, it is not surprising to find such procedures more frequently performed in the ED for non-GLF than for GLF. In this study, the patients in non-GLF had a significantly higher ISS than those in GLF. Such a higher ISS reflects not only the significantly higher incidences of procedures performed at the ED, but also the higher rates of ICU admission and a longer hospital stay (LOS). Notably, in this study, after adjustment by age, sex, and comorbidities, group II and group III patients had significantly higher adjusted odds of mortality than group I patients. However, with the additional adjustment of ISS, although group II did not have significantly higher adjusted odds of mortality than group I patients, group III patients still had significantly higher adjusted odds of mortality than group I patients. These results also imply that even when the severity of injury is strong, it is not the only predictor of mortality across the spectrum of injury mechanisms. Other variables, such as physiological response to the injury, e.g., revised trauma score (RTS, a coded physiological variable values of a patient’s initial GCS score, SBP and RR) [33], and the patients’ health and nutrition status may also play a role in determining the outcome of the patients.

## 5. Limitations

There are some limitations to our study. First, due to the retrospective nature of analysis, some selection bias may exist. Second, patients who died at the scene or those who were pronounced dead on arrival or at the ED were not included, which may lead to a bias in the outcome measurement. The absence of these populations may underestimate the injury severity and mortality from high falls reported in our study. Third, it was not known what type of impact surface or which part of the body was initially affected, and these are also important factors affecting mortality [9]. Moreover, because the kinetic energy of a fall depends on body mass and height [34], the failure to include parameters such as body weight and body mass index may lead to bias in the evaluation, as a previous study [35] has reported that weight and height have an influence on the mortality rate of patients in fall accidents. The causes of fall are multifactorial and complex, particularly those from greater heights. These factors include risky activity (trades requiring working at heights), individual characteristics (demography, educational level), environmental factors, and agents (scaffolds/ladders) [36]. However, our data also did not provide information about the location of fall, impact surface, work status, and activity at time of injury. In addition, there were significantly more intentional injuries in patients with a fall from >6 m. Some bias in the outcome measurement may exist in that data regarding drug-related injuries among patients with suicidal attempts were not included in the registered trauma data. Lastly, this study was performed based on the medical data of one single trauma center and thus it may not be possible to generalize the results to other regions. In the future, a prospective, randomized controlled study would be warranted to estimate the effect.

## 6. Conclusions

This study demonstrated that the height of fall did have impact on mortality in patients with fall accidents. Those patients with non-GLF were more severely injured, had a significantly worse physiological response, and required more resuscitation procedures than those with GLF. A significantly higher adjusted odds of mortality was found in patients with non-GLF than in those with GLF after adjusting for age, sex, and comorbidities.

## Figures and Tables

**Table 1:** Demographics characteristics of patients with falls from heights of < 1 m (m), 1–6 m, and >6 m.

Variables	<1 m		1–6 m		>6 m		1–6 m vs. <1 m			>6 m vs. <1 m		
	(*n* = 7001)		(*n* = 1588)		(*n* = 110)		OR (95% CI)		*p*	OR (95% CI)		*p*
Age, (years)	68.7	±15.6	53.1	±15.3	42.2	±14.7	-		<0.001	-		<0.001
Gender, *n* (%)									<0.001			<0.001
Male	2533	(36.2)	1241	(78.1)	83	(75.5)	6.3	(5.55–7.17)		5.4	(3.50–8.39)	
Female	4468	(63.8)	347	(21.9)	27	(24.5)	0.2	(0.14–0.18)		0.2	(0.12–0.29)	
Intention, *n* (%)	0	(0.0)	15	(0.9)	33	(30.0)	-		<0.001	-		<0.001
Non-intention, *n* (%)	6975	(99.6)	1564	(98.5)	74	(67.3)	0.2	(0.14–0.42)	<0.001	0.01	(0.00–0.01)	<0.001
Comorbidities, *n* (%)												
CVA	794	(11.3)	39	(2.5)	1	(0.9)	0.2	(0.14–0.27)	<0.001	0.1	(0.01–0.51)	0.001
HTN	3658	(52.2)	377	(23.7)	8	(7.3)	0.3	(0.25–0.32)	<0.001	0.1	(0.04–0.15)	<0.001
CAD	569	(8.1)	52	(3.3)	1	(0.9)	0.4	(0.29–0.51)	<0.001	0.1	(0.01–0.74)	0.006
CHF	141	(2.0)	9	(0.6)	0	(0.0)	0.3	(0.14–0.55)	<0.001	-		0.133
DM	1930	(27.6)	230	(14.5)	6	(5.5)	0.4	(0.38–0.52)	<0.001	0.2	(0.07–0.35)	<0.001
ESRD	355	(5.1)	20	(1.3)	0	(0.0)	0.2	(0.15–0.38)	<0.001	-		0.015

CAD = coronary artery disease; CHF = congestive heart failure; CI = confidence interval; CVA = cerebral vascular accident; DM = diabetes mellitus; ESRD = end-stage renal disease; IQR = interquartile range; OR = odds ratio.

**Table 2:** Injury characteristics of patients with falls from heights of < 1 m (m), 1–6 m, and >6 m.

Variables	<1 m		1–6 m		>6 m		1–6 m vs. <1 m			>6 m vs. <1 m		
	(*n* = 7001)		(*n* = 1588)		(*n* = 110)		OR (95% CI)		*p*	OR (95% CI)		*p*
GCS (median, IQR)	15.0	(15.0–15.0)	15.0	(15.0–15.0)	15	(12.8–15.0)	-		<0.001	-		<0.001
3–8	164	(2.3)	94	(5.9)	14	(12.7)	2.6	(2.02–3.40)	<0.001	6.1	(3.40–10.88)	<0.001
9–12	203	(2.9)	67	(4.2)	13	(11.8)	1.5	(1.11–1.96)	0.007	4.5	(2.47–8.14)	<0.001
13–15	6634	(94.8)	1427	(89.9)	83	(75.5)	0.5	(0.40–0.60)	<0.001	0.2	(0.11–0.27)	<0.001
ISS (median, IQR)	9.0	(4.0–9.0)	9.0	(4.0–14.0)	17.5	(9.0–25.0)	-		<0.001	-		<0.001
1–15	6189	(88.4)	1197	(75.4)	46	(41.8)	0.4	(0.35–0.46)	<0.001	0.1	(0.06–0.14)	<0.001
16–24	659	(9.4)	267	(16.8)	35	(31.8)	1.9	(1.67–2.27)	<0.001	4.5	(2.98–6.76)	<0.001
≥25	153	(2.2)	124	(7.8)	29	(26.4)	3.8	(2.97–4.84)	<0.001	16.0	(10.18–25.22)	<0.001
AIS ≥ 2												
Head/Neck, *n* (%)	1098	(15.7)	437	(27.5)	39	(35.5)	2.0	(1.80–2.32)	<0.001	3.0	(1.99–4.39)	<0.001
Face, *n* (%)	185	(2.6)	122	(7.7)	25	(22.7)	3.1	(2.42–3.88)	<0.001	10.8	(6.78–17.33)	<0.001
Thorax, *n* (%)	253	(3.6)	289	(18.2)	52	(47.3)	5.9	(4.96–7.10)	<0.001	23.9	(16.11–35.49)	<0.001
Abdomen, *n* (%)	159	(2.3)	187	(11.8)	50	(45.5)	5.7	(4.61–7.15)	<0.001	35.9	(23.87–53.87)	<0.001
Extremity, *n* (%)	5394	(77.0)	1003	(63.2)	85	(77.3)	0.5	(0.46–0.57)	<0.001	1.0	(0.65–1.59)	0.955
LOS in hospital, (days)	8.2	±8.2	10.6	±11.1	21.2	±16.0	-		<0.001	-		<0.001
ICU admission, *n* (%)	1027	(14.7)	368	(23.2)	64	(58.2)	1.8	(1.53–2.01)	<0.001	8.1	(5.51–11.89)	<0.001
Mortality, *n* (%)	178	(2.5)	56	(3.5)	6	(5.5)	1.4	(1.03–1.90)	0.033	2.2	(0.96–5.10)	0.056
AOR of mortality	-		-		-		2.2	(1.64–2.89)	<0.001	2.5	(1.84–3.38)	0.001
AOR of mortality (ISS)	-		-		-		1.4	(0.95–2.22)	0.082	10.0	(2.22–33.33)	0.002

AOR of mortality = adjusted odds ratio, adjusted by age, gender, and comorbidities; AOR of mortality (ISS) = adjusted odds ratio, adjusted by age, gender, comorbidities, and ISS; CI = confidence interval; GCS = Glasgow Coma Scale; HTN = hypertension; ICU = intensive care unit; IQR= interquartile range; ISS = injury severity score; LOS = length of stay; OR = odds ratio.

**Table 3:** Physiological response of the patients and procedures performed at the emergency department after falls from different height.

Variables	<1 m		1–6 m		>6 m		1–6 m vs. < 1 m			>6 m vs. <1 m		
	(*n* = 7001)		(*n* = 1588)		(*n* = 110)		OR (95% CI)		*p*	OR (95% CI)		*p*
Physiological response at ED, *n* (%)												
GCS < 13	367	(5.2)	161	(10.1)	27	(24.5)	2.0	(1.68–2.48)	<0.001	5.9	(3.76–9.19)	<0.001
SBP < 90 mmHg	57	(0.8)	29	(1.8)	7	(6.4)	2.3	(1.44–3.56)	0.001	8.3	(3.69–18.59)	<0.001
RR< 10 or > 29 beats/min	5	(0.1)	12	(0.8)	4	(3.6)	10.7	(3.75–30.28)	<0.001	52.8	(13.98–199.38)	<0.001
Procedures performed at ED, *n* (%)												
Endotracheal intubation	90	(1.3)	67	(4.2)	16	(14.5)	3.4	(2.45–4.66)	<0.001	13.1	(7.40–23.10)	<0.001
Chest tube insertion	27	(0.4)	35	(2.2)	14	(12.7)	5.8	(3.51–9.65)	<0.001	37.7	(19.16–74.07)	<0.001
Blood transfusion	144	(2.1)	58	(3.7)	27	(24.5)	1.8	(1.32–2.46)	<0.001	15.5	(9.73–24.65)	<0.001

CI = confidence interval; ED = emergency department; GCS = Glasgow Coma Scale; RR = respiratory rate; SBP = systolic blood pressure; OR = Odds ratio.

**Table 4:** Associated injuries in body regions of patients after falls from different heights.

	(*n* = 7001)		(*n* = 1588)		(*n* = 110)		OR (95% CI)		*p*	OR (95% CI)		*p*
Head trauma, *n* (%)												
Cranial fracture	130	(1.9)	131	(8.2)	12	(10.9)	4.8	(3.70–6.10)	<0.001	6.5	(3.47–12.08)	<0.001
Epidural hematoma (EDH)	81	(1.2)	87	(5.5)	10	(9.1)	5.0	(3.64–6.74)	<0.001	8.5	(4.30–16.96)	<0.001
Subdural hematoma (SDH)	654	(9.3)	245	(15.4)	18	(16.4)	1.8	(1.51–2.07)	<0.001	1.9	(1.14–3.17)	0.016
Subarachnoid hemorrhage (SAH)	291	(4.2)	175	(11.0)	19	(17.3)	2.9	(2.35–3.47)	<0.001	4.8	(2.90–8.00)	<0.001
Intracerebral hematoma (ICH)	124	(1.8)	50	(3.1)	5	(4.5)	1.8	(1.29–2.52)	0.001	2.6	(1.06–6.59)	0.049
Cerebral contusion	245	(3.5)	114	(7.2)	7	(6.4)	2.1	(1.70–2.68)	<0.001	1.9	(0.86–4.07)	0.113
Cervical vertebral fracture	35	(0.5)	55	(3.5)	4	(3.6)	7.1	(4.66–10.95)	<0.001	7.5	(2.62–21.51)	0.003
Maxillofacial trauma, *n* (%)												
Orbital fracture	37	(0.5)	23	(1.4)	3	(2.7)	2.8	(1.64–4.67)	<0.001	5.3	(1.60–17.38)	0.024
Nasal fracture	14	(0.2)	18	(1.1)	1	(0.9)	5.7	(2.84–11.53)	<0.001	4.6	(0.60–35.13)	0.209
Maxillary fracture	77	(1.1)	68	(4.3)	11	(10.0)	4.0	(2.89–5.60)	<0.001	10.0	(5.15–19.37)	<0.001
Mandibular fracture	37	(0.5)	20	(1.3)	6	(5.5)	2.4	(1.39–4.15)	0.002	10.9	(4.49–26.29)	<0.001
Thoracic trauma, *n* (%)												
Rib fracture	200	(2.9)	247	(15.6)	34	(30.9)	6.3	(5.15–7.62)	<0.001	15.2	(9.91–23.34)	<0.001
Sternal fracture	1	(0.0)	5	(0.3)	1	(0.9)	22.1	(2.58–189.38)	0.001	64.2	(3.99–1033.36)	0.031
Hemothorax	7	(0.1)	14	(0.9)	7	(6.4)	8.9	(3.58–22.05)	<0.001	67.9	(23.40–197.09)	<0.001
Pneumothorax	24	(0.3)	36	(2.3)	9	(8.2)	6.7	(4.01–11.34)	<0.001	25.9	(11.75–57.13)	<0.001
Hemopneumothorax	23	(0.3)	40	(2.5)	14	(12.7)	7.8	(4.68–13.13)	<0.001	44.2	(22.10–88.59)	<0.001
Lung contusion	7	(0.1)	21	(1.3)	4	(3.6)	13.4	(5.68–31.55)	<0.001	37.7	(10.87–130.73)	<0.001
Thoracic vertebral fracture	67	(1.0)	68	(4.3)	22	(20.0)	4.6	(3.29–6.52)	<0.001	25.9	(15.30–43.76)	<0.001
Abdominal trauma, *n* (%)												
Hepatic injury	6	(0.1)	21	(1.3)	12	(10.9)	15.6	(6.30–38.77)	<0.001	142.8	(52.51–388.07)	<0.001
Splenic injury	4	(0.1)	11	(0.7)	2	(1.8)	12.2	(3.88–38.37)	<0.001	32.4	(5.87–178.74)	0.003
Retroperitoneal injury	2	(0.0)	7	(0.4)	4	(3.6)	15.5	(3.22–74.66)	<0.001	132.1	(23.93–728.81)	<0.001
Renal injury	6	(0.1)	9	(0.6)	2	(1.8)	6.6	(2.36–18.70)	<0.001	21.6	(4.31–108.18)	0.006
Lumbar vertebral fracture	111	(1.6)	150	(9.4)	37	(33.6)	6.5	(5.03–8.33)	<0.001	31.5	(20.31–48.74)	<0.001
Sacral vertebral fracture	11	(0.2)	28	(1.8)	13	(11.8)	11.4	(5.67–22.96)	<0.001	85.2	(37.23–194.83)	<0.001
Extremity trauma, *n* (%)												
Clavicle fracture	101	(1.4)	78	(4.9)	7	(6.4)	3.5	(2.61–4.77)	<0.001	4.6	(2.11–10.23)	0.001
Humeral fracture	407	(5.8)	80	(5.0)	9	(8.2)	0.9	(0.67–1.10)	0.230	1.4	(0.73–2.88)	0.301
Radial fracture	952	(13.6)	282	(17.8)	17	(15.5)	1.4	(1.19–1.59)	<0.001	1.2	(0.69–1.96)	0.575
Ulnar fracture	331	(4.7)	95	(6.0)	10	(9.1)	1.3	(1.01–1.62)	0.040	2.0	(1.04–3.90)	0.034
Metacarpal fracture	58	(0.8)	31	(2.0)	5	(4.5)	2.4	(1.54–3.70)	<0.001	5.7	(2.24–14.50)	0.003
Pelvic fracture	53	(0.8)	90	(5.7)	30	(27.3)	7.9	(5.58–11.11)	<0.001	49.2	(29.84–80.98)	<0.001
Femoral fracture	2777	(39.7)	173	(10.9)	18	(16.4)	0.2	(0.16–0.22)	<0.001	0.3	(0.18–0.49)	<0.001
Tibia fracture	168	(2.4)	81	(5.1)	11	(10.0)	2.2	(1.67–2.87)	<0.001	4.5	(2.38–8.58)	<0.001
Fibular fracture	106	(1.5)	49	(3.1)	8	(7.3)	2.1	(1.47–2.92)	<0.001	5.1	(2.42–10.74)	<0.001
Calcaneal fracture	324	(4.6)	103	(6.5)	12	(10.9)	1.4	(1.14–1.80)	0.002	2.5	(1.37–4.64)	0.006
Metatarsal fracture	89	(1.3)	138	(8.7)	20	(18.2)	7.4	(5.63–9.71)	<0.001	17.3	(10.18–29.25)	<0.001

## Abbreviations

AIS	Abbreviated injury scale
AOR	adjusted odds ratio
CI	confidence interval
CAD	coronary artery disease
CHF	congestive heart failure
CVA	cerebrovascular accident
DM	diabetes mellitus
ED	emergency department
ESRD	end stage renal disease
GCS	Glasgow Coma Scale
GLF	ground-level falls
HTN	hypertension
ICU	intensive care unit
ISS	injury severity score
LOS	length of stay
OR	odds ratio
RR	respiratory rate
RTS	revised trauma score
SBP	systolic blood pressure
